# Promotion of physical activity-related health competence in physical education: study protocol for the GEKOS cluster randomized controlled trial

**DOI:** 10.1186/s12889-019-6686-4

**Published:** 2019-04-11

**Authors:** Stephanie Haible, Carmen Volk, Yolanda Demetriou, Oliver Höner, Ansgar Thiel, Ulrich Trautwein, Gorden Sudeck

**Affiliations:** 1 Institute of Sports Science, University of Tübingen, Wilhelmstr. 124, D-72074 Tübingen, Germany; 2 TUM Department of Sport and Health Sciences, Technical University of Munich, Georg-Brauchle-Ring 60/62, D-80992 Munich, Germany; 3 Hector Research Institute of Education Sciences and Psychology, University of Tübingen, Europastraße 6, D-72072 Tübingen, Germany

**Keywords:** Physical education, Physical literacy, Health-related fitness knowledge, Health literacy, Learning task, RCT, Intervention fidelity

## Abstract

**Background:**

One central goal of physical education in many countries is to empower students to be physically active throughout their lifespan. Physical activity-related health competence (PAHCO) encompasses physical, cognitive, and motivational elements associated with the individuals’ ability to be physically active in a health-enhancing way. To date, there is a lack of empirical evidence concerning effective programs and methods to promote PAHCO in physical education. The purpose of this study is to examine to what extent a health and physical fitness-related program that includes learning tasks integrating theoretical and practical elements promotes students’ PAHCO in physical education.

**Design/methods:**

This study is a cluster randomized controlled trial that compares two physical education intervention programs on health and physical fitness (IG-run, IG-game play) with regular physical education lessons (CG-run, CG-game play) in secondary schools in Germany. Forty-eight physical education classes (ninth grade) were recruited and randomly allocated to the four study groups. The intervention programs include six physical education lessons on health and physical fitness and only differ in the type of physical activity that is executed (running and jumping vs. small-sided games). The students’ PAHCO is examined both pre- and post-intervention and after 8–12 weeks of follow-up. We also determine various process variables during the intervention period to analyze the intervention fidelity.

**Discussion:**

The results of this study provide evidence on whether a combination of theoretical and practical elements in physical education can enhance students’ PAHCO. Beyond that, our process analyses will allow differentiated insights into the mechanism of how the intervention programs work.

**Trial registration:**

German Clinical Trials Register (DRKS), DRKS-ID: DRKS00016349. Retrospectively registered on 10 January 2019.

**Electronic supplementary material:**

The online version of this article (10.1186/s12889-019-6686-4) contains supplementary material, which is available to authorized users.

## Background

The enhancement of students’ knowledge, understanding, skills, and motivation to enjoy a (healthy) physically active lifestyle throughout the lifespan has been acknowledged as a central goal of physical education (PE) in many countries (e.g. [1, 2]). The main goal of the project GEKOS (Förderung bewegungsbezogener Gesundheitskompetenz im Sportunterricht) is to investigate the impact of a health and physical fitness-related PE program that combines practice and theory on physical activity-related health competence (PAHCO) in lower secondary students. In recent years, various school-based intervention studies aimed to promote students’ health or physical fitness, increase their physical activity (PA) level, or affect the psychological determinants of PA (e.g., knowledge, motivation, attitudes towards PA) [3, 4]. According to a review by Demetriou and Höner [5], school-based interventions that include a PA component can significantly affect students’ physical fitness level (70% of the reviewed studies) or PA behavior (57% of the reviewed studies). In addition, this review demonstrated that considerably more intervention studies have examined the effect on physical fitness (51%) or PA (57%) than on different psychological determinants of PA (e.g., knowledge, attitude (12%)). When psychological determinants were investigated, intervention studies reported an entirely positive impact upon students’ knowledge (87%), whereas the effects on other psychological determinants varied between the studies. Moreover, most of the studies did not analyze the intervention effects on PA, physical fitness and health, or psychological determinants at the same time (84%). Further, only 8% of the included studies were rated as high quality methodological studies (e.g., 16% of the reviewed studies included a follow-up measurement to determine long-term effects, only 32% randomized students into different study conditions). In addition, only a minority of studies provided a theoretical foundation (21%, e.g., social-cognitive theory), reported on the quality of intervention delivery, or analyzed the underlying mechanism how the respective effects of an intervention program were evoked (e.g., using a process analysis). However, differentiated analyses of effects and processes are necessary to identify differential effects in subgroups or to be able to accurately interpret possible mechanisms underlying the interventional effects [6, 7]. For example, in a PE health promotion program with strength and endurance exercises, process analyses showed that boys complained about the lack of ball games. Beyond that, they reported that they had less fun in PE during the health promotion program than girls compared with regular PE. The different acceptance of the health promotion program in this study was associated with higher benefits on girls’ than on boys’ physical fitness [3, 8]. These findings raise the question of gender-specific effectiveness of health promotion programs in PE.

Against this background, we designed the current study as a cluster randomized controlled trial with follow-up measurements aiming to promote competences for a healthy, physically active lifestyle. In most Anglo-Saxon PE-curricula, physical, cognitive, and motivational elements associated with a physically active lifestyle are described by the concept of physical literacy (e.g., [1, 9]). At the same time, physical literacy is the main purpose of PE in many countries [10]. PE curricula in Germany (e.g., [11]) aim to achieve the goal of a physically active lifestyle by fostering a variety of different competences (e.g., movement competence) as well as by considering the different values concerning PA as “the value of health and physical fitness”.^1^ In recent years, researchers from different fields have developed competence models dealing with health and physical fitness issues related to PA (e.g., [12, 13]). These models are compatible to physical literacy and health literacy concepts especially in Anglo-Saxon regions representing the intersection of both concepts [14, 15]. In concrete terms, they focus on knowledge, skills, motivation, and abilities considered important to initiate and maintain health-enhancing PA behavior.

The forthcoming trial aligns to the PAHCO model by Sudeck and Pfeifer [13]. It encompasses three sub- competences (movement, control, and self-regulation competence) that are built on different elements among cognitive, physical, and motivational domains. Of these, particularly control competence needs to be highlighted as it plays a central role in being physically active and engaging in PA in a health-enhancing way. Therefore, control competence not only has an effect on the quantity but especially on the quality of PA (e.g., in terms of optimizing health benefits of PA). The mentioned sub-competence can be further divided in two sub-facets focusing on physical health (control competence for physical training) and subjective well-being (control competence for PA-specific affect regulation). This study targets particularly control competence for physical training and the underlying elements of the cognitive domain (health-related fitness knowledge), physical domain (physical fitness) as well as motivational domain (interest and attitudes). Individual control competence for physical training depends on ones understanding of health-related fitness knowledge and its appropriate application to gear PA to individual health. Furthermore, it is related to the ability to be aware of body signals and to use these to control physical load. Summarized, control competence for physical training is not only affected by mere knowledge but also by an understanding and appropriate application of this knowledge in order to adjust actual PA with the goal of promoting health (and well-being) [13]. Hence, a combination of teaching knowledge and PA-related skills and abilities are required for the acquisition of PAHCO in particular for control competence. Studies, which investigated different health behaviors based on the information-motivation-behavioral skills model, support this assumption by demonstrating a relationship between behavior-specific information and behavioral skills that are associated with a specific health behavior [16, 17]. Further, e.g., Fisher and colleagues [18] assumed that in turn the experience of positive health outcomes supports knowledge acquisition and motivation. This finding is in line with pedagogical assumptions that the perception of experiencing training and physical fitness also supports knowledge acquisition and motivation [19].

In Germany, there are examples about how to teach health-related contents in PE [20, 21]. However, there is a lack of research on how to promote PAHCO including not only the improvement of motor skills and abilities but also cognitive-based competence facets.

The linkage of practice (performing PA) and theory is discussed as a constructive method to teach competences in PE [22]. The use of reflective practice, that means conscious reflection-in-action or reflection-on-action, is a particular method to combine theory and practice [23]. In addition, in educational literature, particular learning tasks are attributed to enhance students’ competence. These tasks are cognitively activating, differentiate by students’ ability and facilitate interaction between students [24].

The primary aim of this study is to evaluate the impact of two health and physical fitness-related programs on the acquisition of PAHCO compared to a control group in PE. The two interventions include the same topics (health and physical fitness) as well as methodological concepts and only differ regarding the type of PA that is used to pass on the programs’ content to the students: Running and jumping activities were chosen as a more common type of PA in the context of promoting health and physical fitness, whereas small-sided (ball) games were selected to consider possible gender-specific preferences of PA [3, 25]. Moreover, small-sided games are also appropriate to promote physical fitness and health in youth [26, 27]. Additionally, process analyses are included in the trial to assess the fidelity and quality of intervention delivery, to clarify causal mechanisms, and to identify contextual factors associated with any variations in outcomes [6, 28–30]. Process analyses and intervention programs were developed and tested in two pilot studies (Pilot Study 1, Pilot Study 2) that are also outlined in this study protocol.

### Objectives of GEKOS

Predominantly, GEKOS investigates whether 6-week intervention programs called “run”/“game play” (running and jumping/small-sided ball games with a focus on health and physical fitness) lead to a higher control competence for physical training in ninth grade students as well as of health-related fitness knowledge (cognitive domain) compared to regular PE lessons in control groups “run”/“game play” (running and jumping/games). Additionally, the PAHCO model proposes that there are effects on physical fitness (physical domain) as well as on health and physical fitness-related interest and attitudes (motivational domain). In line with the rationale for the evaluation of complex interventions [28], this study includes multiple outcomes with respect to the acquisition of competence and associated cognitive, physical, and motivational elements.

Further, in accordance with previous study results, it is assumed that the positive effects of the run/game play intervention upon outcomes are moderated not only by gender but also by interest in the run/game play intervention content on the student level, which, in turn, is also assumed to correlate with gender [3, 8].

In line with Fisher et al. [18] and Baschta and Thienes [19], we hypothesize that the effectiveness of the run/game play intervention for control competence for physical training, health related-fitness knowledge (cognitive domain), and health and physical fitness-related interest and attitudes (motivational domain) would be mediated by students’ (perceived) physical fitness.

We use fidelity measures (as part of the process analyses) to investigate how the core components of the run/game play intervention are delivered and as to whether the control group teachers implement the specifications as laid out in the fidelity protocol. This procedure allows us to accurately interpret the treatment effects [6, 7]; the particular aims concerning intervention fidelity are as follows:

- To examine the associations between the quality of the intervention delivery and the outcome measures.
- To investigate the impact of teachers’ attitudes concerning the core components of the run/game play intervention upon the quality of the intervention delivery.
- To determine to what extent students’ perception of the intervention’s core components mediate the intervention effects on the outcomes and also to investigate the moderating role of learning motivation.
- To assess which student characteristics impact student responsiveness (learning motivation, acceptance, and evaluation).

In addition, we analyze the duration and intensity of PA to gain deeper insight into the teaching processes of both the intervention and control groups. This process allows us to investigate the implications of integrating theoretical contents in PE.

## Methods

### Design

**Fig. 1:**
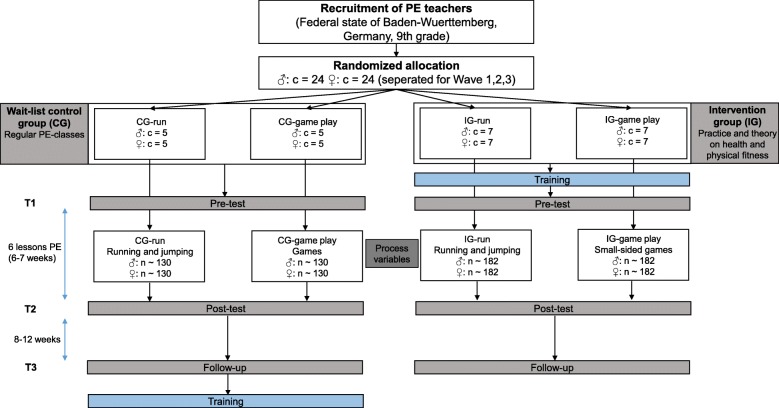
The study design containing the planned number of classes and students prior to the study

The GEKOS study is a cluster randomized controlled trial that includes two intervention groups (IG-run, IG-game play) and two wait-list control groups (CG-run, CG-game play). The study is designed to investigate the superiority of an intervention compared to a control condition. Overall, we recruited 48 ninth grade PE classes and their PE teachers for this study. At this age, boys and girls in the federal state of Baden-Wuerttemberg (Germany) take separate PE classes, so we studied 24 male classes and 24 female classes. We randomly allocated the participating classes to the different study conditions stratified by gender. In order to gain more information about the quality of the intervention delivery, the relative number of classes differed between IG and CG. Accordingly, 14 classes were planned to be allocated to each intervention group prior to the intervention study, whereas 10 classes were to be placed into each control group. Further, teachers were not blinded to the study conditions as they received instructions about the intervention and control conditions prior to the study. Figure 1 illustrates the number of classes (c) and students (*n*) planned to be allocated to the different study conditions.

Over the course of the study, students are tested pre-intervention, post-intervention, and after 8–12 weeks follow-up during their regular school lessons (Fig. 1). The post-intervention and follow-up times may vary for organizational reasons (e.g., school holidays, examinations, canceled lessons). To evaluate the intervention delivery and the students’ duration and intensity of PA, several process variables are assessed during the intervention period. To realize the intervention and comprehensive assessments across 48 classes, the study is carried out in three waves (Wave 1: 1st semester 2017/2018, Wave 2: 2nd semester 2018, Wave 3: 1st semester 2018/2019).

The present study protocol adheres to the Standard Protocol Items: Recommendations for Intervention Trials (SPIRIT) guidelines [31]. The SPIRIT Checklist is provided as supplementary material (see Additional file 1).

### Study setting and participants

Ninth grade PE classes with their respective teachers across secondary schools (Gymnasium) take part in the study. In order to be eligible for the study, PE classes had to fulfill a range of inclusion criteria: the school principal had to approve study participation; PE teachers must have completed their respective degrees; participating students had chosen majors other than PE; and classes had to be located in Baden-Wuerttemberg, Germany.

### Recruitment

The heads of the regional school boards of Baden- Wuerttemberg were the main gatekeepers for recruitment. In preparation for each study wave, we asked the heads of the school boards three to four months in advance to contact the school principal and the teachers who are responsible for PE at their schools (PE subject coordinators) and to invite them to participate in the study. Additionally, the main researchers contacted PE teachers at schools that participated in pilot studies and also former PE students at a University in Germany. During recruitment, interested PE teachers received detailed descriptions of the study and an information sheet to share with their school principals. We informed those who agreed to participate in the study about group allocation as soon as we completed our randomization procedures. These teachers also received informed consent forms for their students and a timetable with the proposed measurement points for the pre-, post-, and follow-up assessments. We used the same recruitment process for all three study waves.

### Sample size

We determined power calculations prior to the main study to determine the optimal sample size using a mixed-procedure in SAS (multi-level model, restricted ML-estimator). The required number of participants was calculated based on the estimated effect upon control competence for physical training and health-related fitness knowledge by considering available data from previous studies. We determined that a difference of d = 0.30 between the intervention (IG-run, IG-game play) and control groups (CG-run, CG-game play) should be identified with ≥80% power and a 0.05 one-sided significance level. If we assume that an average PE class contains 26 students, the study would require at least 10 PE classes in each group (IG-run, IG-game play, CG-run, CG-game play) according to the power analysis. Therefore, we added four additional classes per intervention group to avoid a reduction in statistical power if a class would withdraw from the study.

### Randomization

We randomly assigned participants to either the control (CG-run, CG-game play) or the experimental conditions (IG-run, IG-game play). Due to gender-segregated PE classes in Baden-Wuerttemberg, we conducted separate randomization procedures within the gender strata. This investigation had three study periods (Waves 1–3); therefore, we designed the randomization procedure to be performed in three blocks of 16 PE classes. The allocation ratios differed between these three study periods. In Waves 1 and 3, we randomly and equally allocated both male and female PE classes to the four study groups (1:1:1:1). For Wave 2, we used unequal allocation at a ratio of three (IG-run): three (IG-game play): one (CG-run): one (CG-game play). The randomization took place prior to each study period according to the guidelines of Hutchinson and Styles in 2010 [32]. An independent staff member who was not involved in this study used a computer to generate random numbers for each PE class separated by gender and then sorted the cases according to their random numbers. As a result, the allocation to the different study groups remained concealed to the researchers. Table 1 summarizes the allocation ratios as well as the planned number of female and male PE classes separated by study wave.

**Table 1:** The planned and effective allocation of the study groups separated by gender and wave

	Allocation ratio and planned number of PE classes		Effective allocated number of participating PE classes after randomization (number of participating students)									
			Male					Female				
Wave	IG-run : IG-game play : CG-run : CG-game play	male/female classes	IG-run (n)	IG-game play (n)	CG-run (n)	CG-game play (n)	Total	IG-run (n)	IG-game play (n)	CG-run (n)	CG-game play (n)	Total
Wave 1	1:1:1:1	8/8	2 (24)	2 (30)	2 (24)	2 (33)	8	2 (45)	2 (34)	2 (25)	2 (31)	8
Wave 2	3:3:1:1	8/8	2 (33)	2 (41)	0 (0)	1 (23)	5^a^	3^b,c^ (56)	2^b^ (39)	0^b^ (0)	1 (24)	6
Wave 3	1:1:1:1	8/8	3 (55)	2 (35)	3 (50)	3 (63)	11^d^	2 (42)	3 (57)	3 (51)	2 (45)	10^d^
Sum		48	7 (112)	6 (106)	5 (74)	6 (119)	24	7 (143)	7 (130)	5 (76)	5 (100)	24

^a^As the recruitment rate had been lower than expected, randomization was conducted with five classes and three additional dummy classes to compensate for the absent three male PE classes

^b^After randomization three female classes allocated to IG-run, IG-game play and CG-run canceled their participation. Therefore, they are not considered in the labeled columns

^c^One female PE teacher could be recruited after randomization. She was allocated to the group of PE teacher who canceled her participation at first (IG-run)

^d^In addition to the listed number of PE classes one male and two female dummy classes were added to randomization to guarantee equal allocation ratio

In Wave 2, we were unable to recruit the intended number of PE classes (8 female and 8 male PE classes). Prior to the study period 2, only 8 female and 5 male PE classes indicated their willingness to take part in this study. Therefore, only 13 PE classes were considered in the randomization. In addition, after we had completed the randomization process, three female PE teachers withdrew their participation. To compensate, another female PE teacher who agreed to take part in the study was allocated to the respective study group. Consequently, only 11 of the 16 expected PE classes (5 male and 6 female PE classes) participated in Wave 2. Once Wave 3 began, we recruited 21 PE classes (10 male and 11 female PE classes) to compensate for the absent PE classes in Wave 2 and also to reach the goal of 48 PE classes. To maintain an equal allocation ratio, we added three dummy PE classes (2 male and 1 female classes) for the randomization process. As a consequence, the effective number of PE classes varied between 2 and 3 classes per group. Table 1 shows the final result of the randomization process.

### Intervention

#### Development process of the intervention

**Fig. 2:**
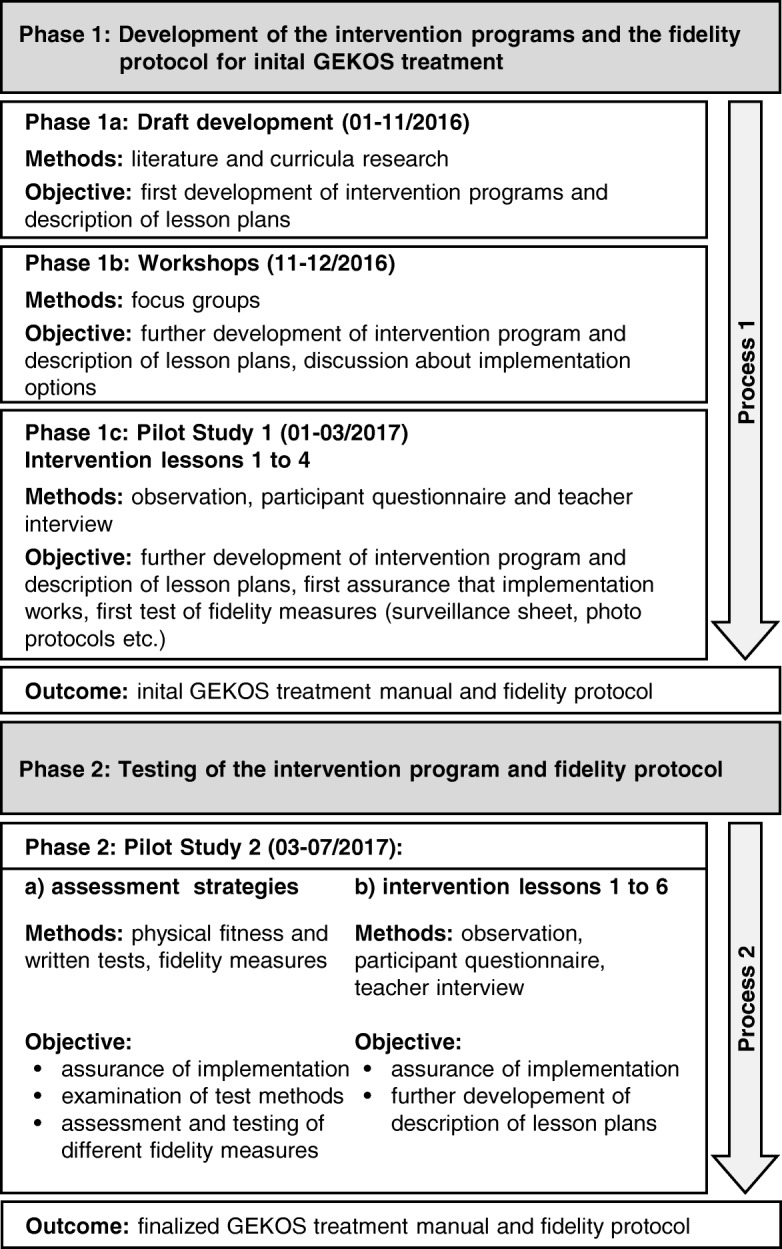
A diagrammatic representation of the development of the finalized treatment manual and fidelity protocol (following Toomey et al. [30])

In Phase 1, which began in January 2016, we started to develop the run/game play intervention for GEKOS and made first drafts of the intervention contents (Fig. 2, Phase 1a). We conducted a literature research regarding health and physical fitness in PE [13] and reviewed the PE-curricula of all German federal states regarding the PAHCO model. In conjunction with teachers and experts in education, we progressed to a first intervention draft for the run/game play intervention. Next, two focus groups discussed both intervention programs with an emphasis on the method, implementation, and comprehensibility of the lesson plans (Phase 1b). In Phase 1c (January 2017), five teachers tested the feasibility of the first four lessons in Pilot Study 1 (2 female and 3 male teachers). At the end of Phase 1, an initial treatment manual was generated.

In Pilot Study 2 (Phase 2), eight teachers (2 females and 2 males per intervention) and their students (*n* = 140) tested the feasibility of the initial manual in practice. We also evaluated the teacher training, assessment strategies (physical fitness and written tests), and fidelity measures (self-report forms for teachers in a checklist style [30] and surveillance sheets).

In both pilot studies, we observed each lesson and assessed these lessons using a formative evaluation, which was later discussed with the teachers in a semi-structured interview. We also asked the students for their opinions using a written questionnaire.

#### Contents of the intervention

The final intervention programs include six PE lessons that are each 90 min in duration. The participating PE teachers of the intervention groups conduct these lessons consecutively within the intended timeframe of 6 weeks. All six PE lessons focus on health and physical fitness and contain the two main topics of the perception of physical load as well as the control of physical load and physical training. In all lessons, students are physically active and also reflect on-action and in-action (“reflective practice”, [23, 33]). Therefore, the lessons include both practical and theoretical elements dealing with the perception and control of physical load as well as the design of a training program to promote health as central aspects of control competence for physical training. The two intervention programs only differ in the type of PA that is carried out during these lessons; the run intervention involves running and jumping activities, while the game play intervention focuses on small-sided games (SSG) and soccer as well as handball drills.

During the development of the intervention programs (Phase 1, Fig. 2), we defined six learning outcomes for the intervention programs, which were used as a framework to define the intervention programs’ content. The intended outcomes for students are as follows:

1. Perform and pace aerobic and muscle strengthening PA.
2. Perceive as well as explain physiological responses to PA (e.g. increasing heart rate) during aerobic or muscle strengthening PA and use the physiological response to PA to regulate PA.
3. Monitor and evaluate acute physiological responses to PA during PA using different techniques (e.g., rating of perceived exertion (RPE)).
4. Name and explain the basic principles of exercise to plan an exercise program.
5. Identify different types of PA and adjust components of the FITT formula (frequency, intensity, time, and type) to affect cardiovascular endurance and muscular endurance while considering a specific objective.
6. Name the effects of PA on health and reflect upon the importance of PA for one’s own health.

**Fig. 3:**
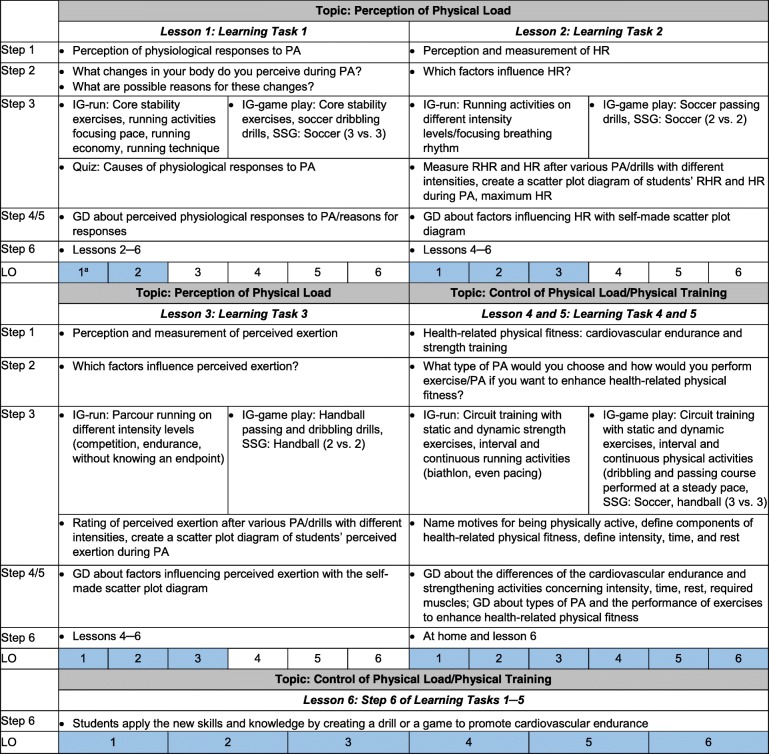
Main topics and learning tasks of the six PE lessons. ^a^ Learning outcomes and their numbers are illustrated in the text. *LO* learning outcome, *GD* group discussion, *HR* heart rate, *RHR* resting heart rate

To support students’ progress concerning the learning outcomes, we developed five learning tasks to guide the six PE lessons. These five tasks cover the two main topics of the perception of physical load as well as the control of physical load and physical training. Each of the five learning tasks includes six steps (Fig. 3), which align with the teaching and learning process for the promotion of competences as described by Leisen [34] that is comparable to the instructional method of problem-based learning [35]. For each step of the learning tasks (except Step 1), we included subtasks for the students to solve consecutively. After presenting the issue (Step 1: e.g., physiological responses during PA), the teachers invite their students to formulate assumptions on the topic (Step 2: e.g., assumptions about changes in the body during PA) without receiving immediate feedback. Subsequently, the students receive information on the topic while solving tasks that combine PA and cognitive elements by reflecting on-action (PA) or in-action (PA; Step 3). During Steps 4 and 5, the students discuss the given information and findings in relation to the lessons’ topic with their respective teacher (Step 4), thus, identifying right and wrong assumptions, and defining relevant outcomes (Step 5). Finally, the teachers ask the students to apply the new skills and knowledge (Step 6; see Fig. 3 for a detailed description of the tasks). All subtasks incorporate aspects considered important for the acquisition of competences in education, such as cognitive activation, student-orientation, and support social-interaction [24].

### Control group

PE teachers in the control group teach six consecutive regular PE lessons focusing on running and jumping activities (CG-run) or on games (CG-game play). We did not give them any guidelines concerning the methods or specific content (except physical activities) of their PE lessons. After the follow-up tests, control group teachers will have the opportunity to attend the same two-hour training session that teachers in the intervention received beforehand. We also will make the manual and materials of one of the intervention programs available to these control group teachers after the study has concluded.

### Measures

**Fig. 4:**
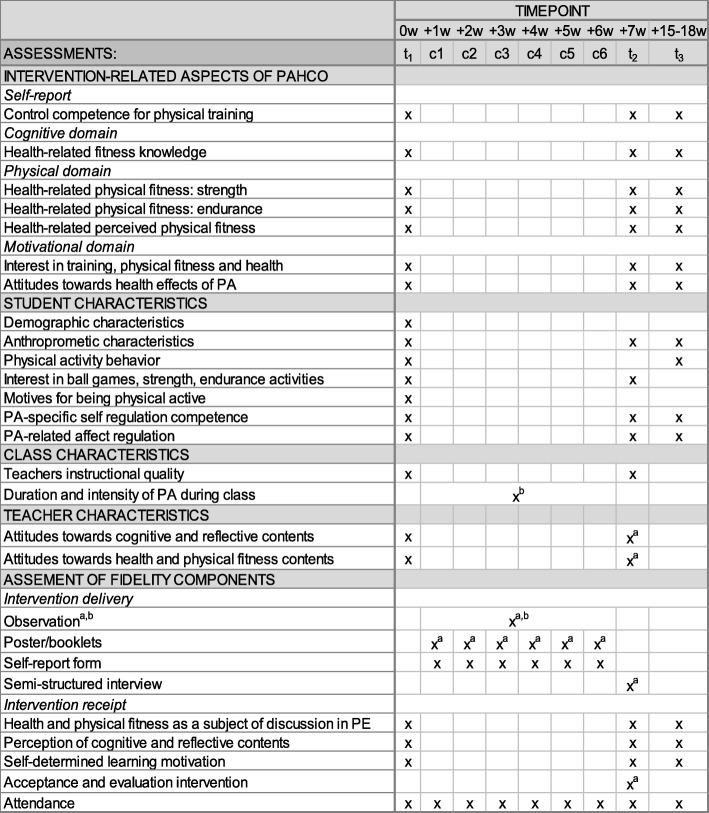
Measurements at each time point: baseline, post-intervention, 8–12 week follow-up, and process measures during the 6-week sessions (following SPIRIT template [31]). ^a^ administered in intervention group only. ^b^ once in each class covering each time point at least once. *w* week; *t* time point; *c* class

In order to test, revise, and optimize all measures before the main study, we applied the scales and physical fitness tests in Pilot Study 2 (Fig. 2). Furthermore, we tested all measurements (except for the physical fitness test) to assess the intervention-related aspects of PAHCO as well as the health and physical fitness as a subject of discussion in PE scale in a sample of 800 students in 2015 prior to beginning the pilot studies. Figure 4 shows a list of measurements of the main study at each time point.

#### Intervention-related aspects of PAHCO

To measure the effect of the intervention programs on control competence for physical training, health-related fitness knowledge, physical fitness, and health and physical fitness-related interest and attitudes, the following validated measures are applied.

### Control competence for physical training

A self-report measurement [13] is used to assess control competence for physical training. This measure consists of six items with responses ranging from strongly disagree (1) to strongly agree (5).

### Cognitive domain

To assess the health-related fitness knowledge, a questionnaire including a performance test is administered to the students. This questionnaire contains 27 complex multiple choice, matching, and short answer items that we developed and validated in-house.

### Physical domain

The physical domain is assessed by examining physical fitness, which includes three strengthening exercises (standing long jump, push-ups, and sit-ups) that are part of a standardized physical fitness test (Deutscher Motorik Test, German motor test) [36] and an endurance test (shuttle run) [37]. During the shuttle run, the maximum heart rate is measured with a heart rate monitoring system (acentas Herzfrequenz Monitoring™ [38]). Furthermore, we measure perceived physical fitness using the physical fitness subscale (four items: well-trained, vigorous, fit, and strong) of the self-assessment scale for perceived physical state (PEPS) [39]. Students provide their responses on a 6-point Likert scale with a range from 0 (not at all) to 5 (strongly).

### Motivational domain

In order to determine the motivational domain, participants provide information regarding their interest in training, physical fitness and health as well as their affective and cognitive attitudes towards the health effects of PA. To operationalize interest, we have developed four items based upon PISA 2006 [40]. Accordingly, we use a questionnaire (four and three items) to assess their attitudes; previous studies have used this questionnaire in adolescents (e.g., Demetriou) [8]. For all scales, students choose from five possible responses on a Likert scale ranging from 1 (strongly disagree) to 5 (strongly agree).

#### Student characteristics

Students report their gender and month of birth on a questionnaire. Standing height and weight are assessed with a regular scale and a portable weight scale and are used to calculate body mass index (BMI). The BMI is then converted into BMI percentiles based on German reference data [41]. Standing height and weight are also necessary to evaluate accelerometer data obtained when measuring activity during PE classes.

Moreover, we capture self-reported PA behavior using the Physical Activity, Exercise, and Sport Questionnaire (BSA- F: derived from German: Bewegungs- und Sportaktivität Fragebogen) [42]. The BSA-F recalls exercise and sport activities that are usually undertaken and asks for type of PA, frequency per week, and duration of each individual PA. We slightly modified the main question from the original scale in order to gain more differentiated insight in the PA behavior of adolescents with regard to PA in sports clubs and during leisure time.

To operationalize motivational factors and further aspects of PAHCO, we use a five-point Likert scale ranging from 1 (strongly disagree) to 5 (strongly agree), to assess the following:

- Interest in ball games (3 items), interest in strength training (3 items), and interest in endurance activities (3 items) developed following PISA, 2006 [40].
- Motives for being physically active (10 items) [36].
- Control competence for PA-related affect regulation (4 items) [13].
- PA-specific self-regulation competence (3 items) [13].

#### Class characteristics

As a potential control variable, we also ask students to evaluate their teachers’ instructional quality on a five-point Likert scale (ranging from strongly disagree to strongly agree) following general and PE-specific validated questionnaires [43–45]:

- Classroom management: monitoring (2 items); discipline (2 items); goal orientation (2 items).
- Student orientation: supportive climate (2 items); motivation (2 items).

During PE classes, we provide an objective measurement of the duration and intensity of PA once in each class (IG and CG) using validated accelerometer sensors (Move III sensor, movisens GmbH, Karlsruhe Germany), which have also been used in previous studies [46–48]. The sensors triacially measure PA; students wear these sensors attached to their right hips with a clip during class.

#### Teacher characteristics

In order to measure teachers’ attitudes towards core components of the intervention (contents and methods), we administer a questionnaire to all teachers. In the questionnaire, we assess attitudes towards cognitive and reflective contents in grade level 9 in PE (adaption following Rischke, 2009 [49]; 6 items) and attitudes towards health and physical fitness contents in grade level 9 (an adaption of the student-scale, health and physical fitness as a subject of discussion, developed following Hoffmann [50] and further extension considering the curricula contents of Baden-Wuerttemberg, Germany; 7 items). For the first scale, we provide six possible responses on a Likert scale ranging from 1 (not important) to 6 (very important). For the second scale, participants choose from five possible responses on a Likert scale ranging from 1 (strongly disagree) to 5 (strongly agree). In addition, a semi-structured interview is conducted with intervention group teachers in which their attitudes regarding the intervention are further explored after the intervention.

#### Assurance and assessment of fidelity components

To assure that the core components of the intervention are implemented as planned [6, 7], we developed a (implementation) fidelity protocol parallel to the treatment manual following Toomey and colleagues [30] (Fig. 2). The fidelity protocol entails techniques, methods, and process measures to provide, ensure, and record fidelity information on intervention design, teacher training, intervention delivery, and intervention receipt during the main study [7, 29, 30]. We developed and optimized these methods and techniques in our two pilot studies (Fig. 2) and partially followed the experience gained in a former intervention study in a school context [8]. Furthermore, the pilot studies supported the identification of confounding factors and factors to improve the quality of intervention delivery by increasing teachers’ acceptance of and positive attitudes towards the interventions.

### Intervention design

The fidelity component, intervention design, ensures that the study can be replicated and evaluated in relation to its underlying theory [7, 29]. Thus, we precisely describe both treatments in the treatment manual along with the theoretical model of PAHCO and the theoretical basis of the tasks. The treatment manual includes an exact description of the interventions, equipment, and materials needed for the intervention and the self-report forms for the intervention group. Furthermore, control group teachers also receive a standardized process description (including self-report forms) for their lessons. Since the treatment and control conditions take place during regular PE lessons, we assume that the dose is the same within conditions and also equivalent across conditions.

### Teacher training

During the main study, the intervention group teachers participate in a standardized training right before the start of the intervention. The training takes place at the teachers’ schools to reduce expense and increase their compliance. During the two-hour training session, teachers receive information about intervention theory, the goals of the study, and the contents of the intervention, including its special features and troubleshooting. All intervention group teachers undertake the same training with the same two main researchers, but their individual experiences and attitudes also factor into the process; every training session is limited to one to three teachers in order to ensure best possible learning outcomes. During the training, the researchers focus especially on the structure of PE lessons in the run/game play intervention and also regularly provide support in the treatment manual trying to avoid drift over time as the process is new to many of the teachers.

### Intervention delivery

Monitoring of the intervention delivery is considered as the heart of fidelity [7]. To ensure adherence to the treatment manual at the class level, we use the following process measures: First, two observers conduct one announced observation per class that focuses on the intervention delivery and assesses performance using standardized surveillance sheets. Second, the teachers send posters with the results of each lesson (students’ assumptions and defined outcomes of each lesson: Steps 2, 4, and 5 of the learning tasks) in the form of a photo protocol. Third, after the intervention, student booklets provide insight into the students’ completion of intended worksheets during class. Fourth, intervention group teachers fill out the self-report form which assesses intervention delivery of the different steps of the learning process, any deviations, and potential incidents. Control group teachers also complete a self-report form to record their class contents. This process indicates whether both intervention and control group teachers do what is expected and helps to identify differences between those groups. After the completion of the intervention, we conduct also a semi-structured interview that focuses on intervention delivery and optimization of implementation. In cases where disruptions occur during the intervention delivery, the teachers’ instructional quality is rated during the observation by the observers (classroom management, student orientation, positive learning environment [43–45]).

### Intervention receipt

Further, we document the intervention receipt to assess whether participants receive the treatment, to determine whether the participants comprehend and use the treatment during the session, and to identify the extent to which participants are engaged in the content of the treatment [7].

We also assess students’ perception with regard to core components of the intervention as follows [43, 50, 51]:

- Health and physical fitness as a subject of discussion in PE (contents, 6 items) [50].
- Perception of cognitive and reflective contents (methods): Cognitive activation (3 items) [43]; methodical reflection (3 items) [51].

We determine student responsiveness by their self-determined learning motivation and also their acceptance and evaluation of the intervention as follows [8, 52]:

- Self-determined learning motivation: self-determined interest (3 items) and self-determined identified learning motivation (3 items) [52].
- Acceptance and evaluation of the intervention: Evaluation of health and physical fitness-related intervention programs; items following the evaluation of HealthyPEP [8], tested and optimized in Pilot Study 2 2017; (8 items and 2 short answer questions).

Participants are asked to provide their responses using a five-point Likert scale ranging from 1 (strongly disagree) to 5 (strongly agree). In addition, to examine whether students receive the intervention as intended, the teachers maintain an attendance list.

### Data collection and management

Trained researchers are expected to follow standard operating procedures to collect and enter data. The operational research team consists of two main researchers, a small group of researchers who support organization and process measures, and a group of research assistants who conduct any other necessary assessments and data entry. Within the test manual all standard operating procedures are documented in great detail and prepared documentation forms are used to describe all incidents and deviations from the test manual during data collection. Additionally, teachers regularly send their photo protocol of the posters digitally after each class to the main researchers. Teachers provide the attendance lists as well as the self-report forms and the students’ booklets after the intervention. These booklets are then scanned and sent back to the teachers so that students can keep their booklet and results acquired during the PE lessons. The same two main researchers conduct and record the interviews with the intervention group teachers. We use F4transkribt to transcribe the interviews. We transfer the quantitative data into a secured electronic database (IBM SPSS Statistics) and double- check the information. We also monitor existing data of all measurements anonymously for students and teachers in separate files. The research assistants (*n* = 27) who conduct data collection at three time points and perform the data entry are not informed about the respective group allocation. However, we cannot guarantee blinding throughout the entire study due to data processing and communication between test coordinators and teachers during the assessments.

### Data analysis

Statistical analysis will be conducted, analyzed, and reported according to the CONSORT statement for cluster RCTs [53, 54]. The overall analytic structure uses a structural equation modeling framework with student level variables and class level variables considering the special feature of nested data (e.g. Mplus: TYPE = COMPLEX). We will use a descriptive analysis to establish recruitment and dropout rates and the distribution of baseline characteristics and all outcomes post-intervention and at the 8–12 week follow-up.

The main analysis involves two a priori comparisons; both intervention groups (run/game play) will be compared to their control groups (run/game play). The analysis will estimate differences at the post-test and during follow-up between the two trial arms (IG-run vs. CG-run /IG-game play vs. CG-game play) after adjusting for baseline data. We will examine these differences using linear regression models with control competence for physical training and domains (cognitive, physical, motivational) as outcomes, the respective pretests as a covariate, and an IG-dummy as a predictor.

For the purpose of a sensitivity analysis, we will compare outcomes either based on the intention-to-treat (ITT) principle with classes (clusters) and students analyzed according to the study groups, or on complier average causal effects [55], which has already been used in prior studies [56]. We also will compare the differences between intervention groups. A multiple group analysis will enable us to estimate the average effects for the run/game play intervention and will allow us to test differences between the run and game play groups.

Further, we will conduct a moderation analysis (multiple group model) that accounts for gender and interest in strength and endurance activities, and respectively, in ball games.

We will carry out a mediation analysis to evaluate the effect of the intervention on physical fitness (strength and endurance) as well as on perceived physical fitness and to what extent this influences the efficacy of the interventions on the cognitive and motivational domain as well as on control competence for physical training.

As a customary practice in longitudinal analysis, it is assumed that there are co-correlated residuals over time. Therefore, model comparisons of models with correlated residuals versus models without such correlations are planned.

In general, we expect unsystematic missing data due to absence of students (illness, etc.) on test days. We will analyze systematic occurring missing data with a lost-to follow-up analysis (students) and with the teacher interview. Any “missing at random” data will be replaced using multiple imputation methods. We will report the amount of missing data as a percentage of the main outcomes along with the data recovered in the imputation analysis.

## Discussion

This study predominantly investigates the effect of the health- and physical fitness-related run/game play intervention in PE on control competence for physical training, health-related fitness knowledge (cognitive domain), physical fitness (physical domain), and health and physical fitness-related interest and attitudes (motivational domain) compared to regular PE in ninth grade students.

The findings of this study have the potential to provide valuable information on whether this special approach to PE (the application of learning tasks to combine theory and practice in PE) promotes the acquisition of PAHCO in adolescents, and - if so - by which indicators the acquisition is moderated.

However, the study is limited in that the second wave did not meet the planned amount of classes. Moreover, the individual classes did not contain the initially expected number of students.

Strengths of this study include the cluster randomized controlled trial design with baseline, post-intervention and follow-up, the detailed quantitative and qualitative process evaluation, and the structured development process of the intervention programs and their extensive pilot testing. This study provides more evidence and insight into intervention delivery and outcomes and allows the evaluation and replication of the GEKOS study. In addition, we are conducting a variety of measures (performance test, physical fitness test, heart rate) and self-report procedures, which also support outcome interpretation.

### Trial status

Data collection in wave 3 began during the first semester of the 2018/2019 academic year and will continue until April 2019; we have already completed recruitment, randomization, and baseline measures.

## Additional file

Additional file 1: The SPIRIT Checklist is provided as supplementary material. (PDF 169 kb)

## Abbreviations

CG: Control group
GD: Group discussion
GEKOS: Promotion of physical activity-related health competence in physical education (Förderung bewegungsbezogener Gesundheitskompetenz im Sportunterricht)
IG: Intervention group
PA: Physical activity
PAHCO: Physical activity-related health competence
PE: Physical education
RPE: Rating of perceived exertion
SSG: Small-sided games

## Footnotes

1 In consideration of German PE research traditions and discussions, which is mostly published in German, we refer to the German literature and attempt to draw connections to English literature in the following sections.
